# High Prevalence of *Haemophilus ducreyi* Among Patients With Suspected Primary syphilis in Malawi, 2019–2022

**DOI:** 10.1093/cid/ciaf114

**Published:** 2025-03-17

**Authors:** Mitch M Matoga, Jane S Chen, Arlene C Seña, Beatrice Ndalama, Naomi Nyirenda, Naomi Bonongwe, Esther Mathiya, Blessings Kamtambe, Edward Jere, Gabriel K Banda, Isaac Thengolose, Jenny Coelho, Christopher M Hennelly, Daniel Schröder, Magnus Unemo, Juan C Salazar, Justin D Radolf, Jonathan B Parr, Irving F Hoffman

**Affiliations:** Reproductive and Sexual Health Clinic, University of North Carolina Project Malawi, Lilongwe, Malawi; Institute of Global Health and Infectious Diseases, University of North Carolina at Chapel Hill, Chapel Hill, North Carolina, USA; Institute of Global Health and Infectious Diseases, University of North Carolina at Chapel Hill, Chapel Hill, North Carolina, USA; Reproductive and Sexual Health Clinic, University of North Carolina Project Malawi, Lilongwe, Malawi; Reproductive and Sexual Health Clinic, University of North Carolina Project Malawi, Lilongwe, Malawi; Reproductive and Sexual Health Clinic, University of North Carolina Project Malawi, Lilongwe, Malawi; Reproductive and Sexual Health Clinic, University of North Carolina Project Malawi, Lilongwe, Malawi; Bwaila STI Clinic, Bwaila District Hospital, Lilongwe District Health Office, Lilongwe, Malawi; Reproductive and Sexual Health Clinic, University of North Carolina Project Malawi, Lilongwe, Malawi; Reproductive and Sexual Health Clinic, University of North Carolina Project Malawi, Lilongwe, Malawi; Reproductive and Sexual Health Clinic, University of North Carolina Project Malawi, Lilongwe, Malawi; Institute of Global Health and Infectious Diseases, University of North Carolina at Chapel Hill, Chapel Hill, North Carolina, USA; Institute of Global Health and Infectious Diseases, University of North Carolina at Chapel Hill, Chapel Hill, North Carolina, USA; Department of Laboratory Medicine, Faculty of Medicine and Health, Örebro University Hospital, Örebro, Sweden; WHO Collaborating Centre for Gonorrhea and Other STIs, Örebro University, Örebro, Sweden; Department of Laboratory Medicine, Faculty of Medicine and Health, Örebro University Hospital, Örebro, Sweden; WHO Collaborating Centre for Gonorrhea and Other STIs, Örebro University, Örebro, Sweden; Connecticut Children's Medical Center, Hartford, Connecticut, USA; Department of Pediatrics, University of Connecticut Health, Farmington, Connecticut, USA; Connecticut Children's Medical Center, Hartford, Connecticut, USA; Department of Pediatrics, University of Connecticut Health, Farmington, Connecticut, USA; Institute of Global Health and Infectious Diseases, University of North Carolina at Chapel Hill, Chapel Hill, North Carolina, USA; Reproductive and Sexual Health Clinic, University of North Carolina Project Malawi, Lilongwe, Malawi; Institute of Global Health and Infectious Diseases, University of North Carolina at Chapel Hill, Chapel Hill, North Carolina, USA

**Keywords:** genital ulcer disease, chancroid, *Haemophilus ducreyi*, primary syphilis, Malawi

## Abstract

**Background:**

As syphilis rates have increased globally, chancroid has dramatically declined as a cause of genital ulcer disease (GUD).

**Methods:**

We recruited patients aged ≥18 years presenting to a sexually transmitted infection clinic with GUD from Lilongwe, Malawi, from November 2019 through April 2022. Lesion exudates were tested by darkfield microscopy (DFM) and polymerase chain reaction (PCR) for *Treponema pallidum* (TP) and by PCR for *Haemophilus ducreyi* (HD), herpes simplex virus, and *Chlamydia trachomatis*. We evaluated the sensitivity and specificity of DFM relative to TP PCR, the distribution of GUD etiologies by PCR, and the performance of our HD PCR relative to Allplex Genital Ulcer assay (Seegene Inc) using the Cohen's kappa statistic.

**Results:**

We enrolled 568 participants; the median age was 27 years (interquartile range: 23, 34), 61% (345/564) were men, and 13% (60/464) had human immunodeficiency virus (HIV) or were newly diagnosed with HIV. DFM identified TP in 55 (10%) participants, with a sensitivity and specificity of 12% and 94%, respectively. PCR identified TP in 367 (65%), HD in 128 (23%), herpes simplex virus in 98 (17%), and *Chlamydia trachomatis* in 36 (6%) of participants with only 1/36 (2.8%) with serovar L1, L2, or L3 consistent with lymphogranuloma venereum; no etiology was identified in 48 (8%). External validation confirmed the high HD prevalence (Cohen's kappa 0.78, 89% agreement).

**Conclusions:**

Syphilis and chancroid are common etiologies of GUD in Malawi. Our findings underscore the value of highly sensitive molecular diagnostic methods to periodically assess GUD causes among patients with sexually transmitted infections in countries using syndromic management.

## Teaser

Although syphilis rates have increased globally, chancroid has dramatically declined as a reported cause of genital ulcers. We found that syphilis and chancroid were common etiologic agents among patients presenting with genital ulcers to a sexually transmitted infection clinic in Lilongwe, Malawi.

Sexually transmitted infections (STIs) in most low-middle income countries (LMICs) are treated using syndromic management [1,2]. Among the STI syndromes, genital ulcer disease (GUD) is common in Malawi [3]. Prior GUD studies found that the most frequent etiologies are *Treponema pallidum* subspecies *pallidum* (TP), *Haemophilus ducreyi* (HD), and herpes simplex virus (HSV) [4–6]. Consequently, Malawi's syndromic GUD therapy still includes treatment for all these pathogens, although World Health Organization (WHO) guidelines no longer recommend empiric chancroid treatment since HD has declined globally [1].

There have been varying trends in GUD etiologies reported in Malawi [4–8]. The proportion of specimens with TP was 18% [5] in 1994, then declined [6] to 5% in 2010 [4,7]. Recently, a pilot study found a notably higher proportion (60%) of TP among GUD cases [9]. Presumably due to its association with HIV prevalence, the proportion of GUD resulting from HSV peaked at 72% in 2013 [4] then decreased to 26% in 2021 [9]. HD was found in 26% of GUD cases in 1994 [5] but declined to 15% in 2013 [4] Although Chen et al [9] reported that 18% of GUD cases in Malawi had HD, their study involved only 50 participants. Last, *lymphogranuloma venereum* (LGV) has contributed 6% of GUD cases in Malawi since the early 2000s [4].

Globally, the epidemiology of chancroid has not been well described. The proportion of GUD caused by HD has ranged from 0% in several African countries to 15% in Malawi [10]. There has been a sustained reduction in the proportion of GUD caused by chancroid over the past 2 decades. However, there has been growing evidence that HD causes nonsexually transmitted skin ulcers in children in LMICs [10].

Darkfield microscopy (DFM) is a specific, direct detection technique for visualizing TP from anogenital lesions [11]. However, DFM is technically challenging and requires specialized equipment. For many STIs, molecular tests have become a preferred method because of their high sensitivities and specificities. Although polymerase chain reaction (PCR)-based testing for GUD pathogens has been limited, nucleic acid amplification tests are available for HSV and syphilis detection [12].

We conducted a study to screen participants with GUD presenting to an STI clinic in Lilongwe, Malawi, as part of a larger study to evaluate the genomic diversity of TP for vaccine development [13]. For this study, we analyzed data from participants who presented with GUD to (1) compare the performance of DFM relative to TP quantitative PCR (qPCR) and (2) conduct molecular detection for other STIs among both DFM-positive and DFM-negative cases.

## MATERIALS AND METHODS

### Study Population

We recruited patients ≥18 years of age presenting with wet/moist anogenital lesions at Bwaila District Hospital STI Clinic in Lilongwe, Malawi, from November 2019 to April 2022. Patients were eligible for screening if they had suspected primary syphilis (PS) and were willing to undergo HIV testing if status was unknown [13]. This study was approved by the institutional review board at the University of North Carolina at Chapel Hill (UNC; IRB# 19-0311) and the Malawian National Health Science Research Committee.

### Screening Procedures

Following written informed consent, participants underwent screening that included collection of basic clinical data (ie, demographics, HIV status) and lesion swabs for DFM. After recognizing that some participants with genital lesions had secondary syphilis (SS), patients with suspected SS were also eligible for screening after February 2022, and rapid point of care (POC) treponemal antibody testing (Alere Determine Syphilis Tp, Abbott) was used to assist with laboratory confirmation. The screening consent also included PCR testing for other STIs on the same lesion swab, so all participants screened had stored specimens for subsequent PCR testing.

As part of routine care, all participants received parallel rapid HIV testing with Alere Determine HIV-1/2 and Uni-Gold HIV. For positive rapid HIV tests, additional testing with SD Bioline HIV-1/2 was conducted for confirmation.

### Darkfield Microscopy Procedures

After cleansing the lesion with PCR-clean water or gauze, lesion exudate was collected using a sterile (Dacron or polyester flocked) swab and transferred to a glass microscope slide, followed by application of a cover slip and sealing the edges to prevent drying during transport to the laboratory (<200 m away). The swab was subsequently placed in a 0.5-mL microfuge tube containing 250-µL of Zymo DNA/RNA Shield.

DFM was conducted within 20–30 minutes by 2 trained laboratory technicians, with initial visualization using 10× and 40× objective lens on a darkfield microscope. Following location of a spirochete, 40× and 100× objectives were used to view the slide in a systematic manner. If there was a discrepancy in the DFM interpretation, the specimen was reviewed in a blinded manner by a third laboratory technician to establish the final result.

### Additional Procedures for Participants With Confirmed Syphilis

Participants confirmed with PS or SS with a positive DFM and/or a positive POC test underwent additional study procedures for syphilis characterization [13]. These participants provided written informed consent for clinical data collection, physical examination, and photography of lesions [13]. Blood samples were collected for Rapid Plasma Reagin (RPR) and TP particle Agglutination Assay (TPPA) testing. Their clinical/laboratory data and lesion photographs were reviewed to confirm syphilis staging.

### GUD Treatment

Following study procedures, all participants with GUD were treated syndromically per the Malawian standard of care, with benzathine penicillin 2.4 MU intramuscularly, ciprofloxacin 500 mg orally twice daily for 3 days, and acyclovir 800 mg orally 3 times per day for 2 days [2]. All participants with HIV infection were referred for care and antiretroviral therapy.

### Molecular Diagnostic Testing

DNA was extracted from all swabs and stored at −80 °C before shipment to the Infectious Disease Epidemiology and Ecology Laboratory at UNC-Chapel Hill, NC, USA. At UNC, quantitative TP *polA* PCR (qPCR) was conducted on samples as previously described [14]. In addition, singleplex PCRs for HD, HSV1/2, and *Chlamydia trachomatis* (CT) were performed using published assays (see Supplementary Material).

Samples with >0 copy numbers by qPCR were considered positive for TP; samples were considered positive for HD, HSV, or CT if the PCR cycle threshold was >0 and <40. To determine CT serovars, a nested segment of the major outer membrane protein gene (*ompA*) was amplified for CT-positive samples [15]. Amplicons underwent bidirectional Sanger sequencing at Eton Bioscience (Research Triangle Park, NC, USA). Consensus sequences will be deposited in NCBI (accession numbers in Supplemental Material) (Supplementary Tables 1 and 2).

### Confirmation of *H. ducreyi* and Other STIs

A subset of samples was shared with the WHO Collaborating Centre for Gonorrhea and other STIs in Örebro, Sweden, for external validation, which included: (1) all HD positive samples; (2) all CT positive samples; and (3) all samples that were negative for pathogens tested. To externally validate the HD and CT results, the WHO Collaborating Centre used the commercially available Allplex Genital Ulcer real-time PCR assay (Seegene Inc, Seoul, South Korea) assay [16]. The Allplex PCR also provided results for TP, HSV, LGV-causing CT strains, and other pathogens, although sample selection was not designed to evaluate the performance of these assays.

### Statistical Analysis

Statistical analysis was limited to all screened participants with GUD and suspected PS/SS with DFM and PCR results. We used descriptive statistics to summarize demographic characteristics, calculated the distribution with TP, HD, HSV, and CT as percentages, and stratified by sex and HIV status. We calculated sensitivity and specificity of DFM relative to TP qPCR results and evaluated the performance of our HD PCR assay using the Cohen's kappa statistic. All analyses were performed using SAS v9.4 (Cary, NC, USA) or R v.4.2.2 (Vienna, Austria).

## RESULTS

### Demographic Characteristics of Study Participants

**Figure 1.**
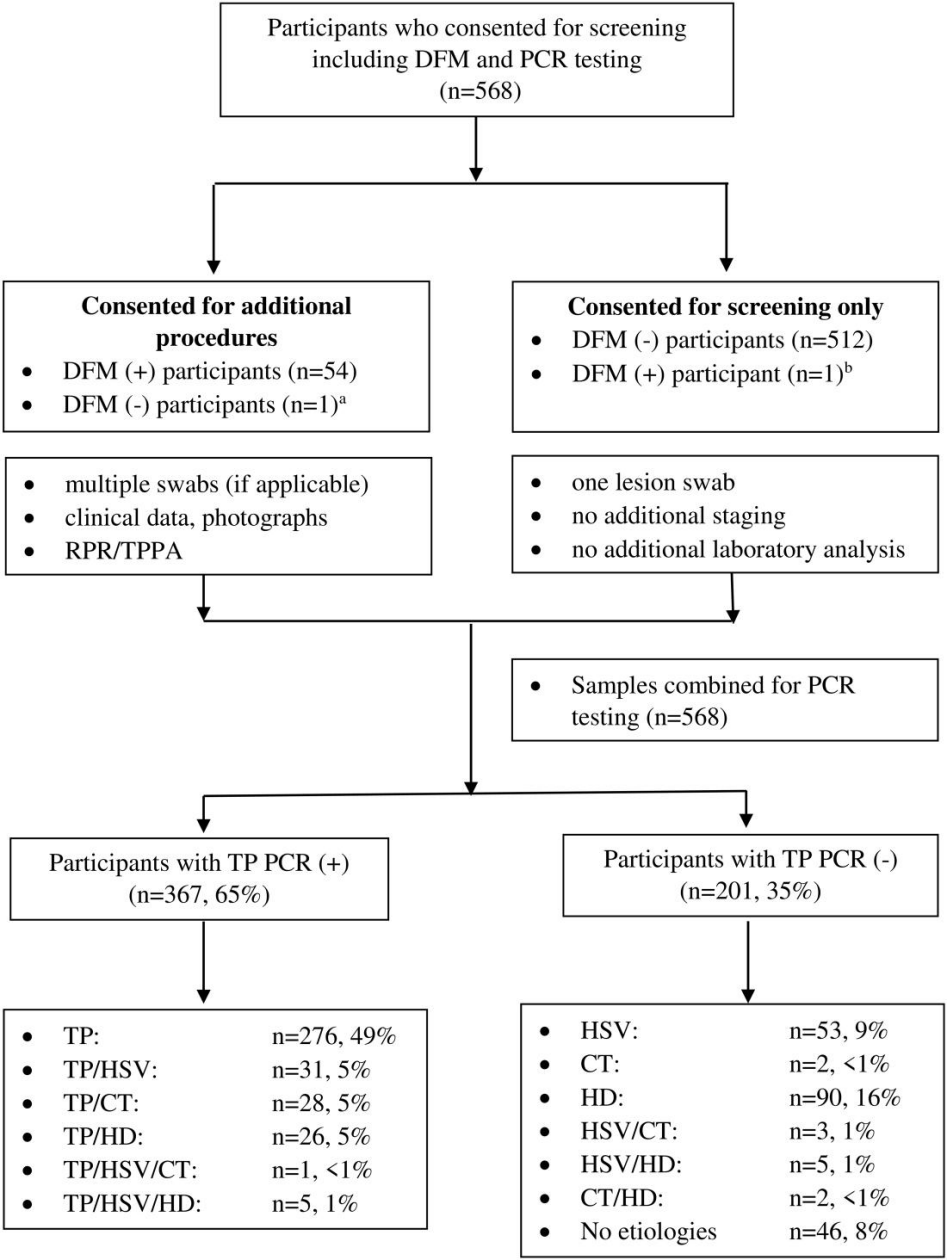
Flow algorithm of participants screened and enrolled in the study with genital ulcer disease and their sexually transmitted infection etiologies.

During the study period, 568 participants with GUD provided consent for screening (Figure 1). The median age was 27 years (interquartile range: 23, 34); 345 (61%) of the 564 participants with documented sex were men. Sixty (13%) of 464 participants who had HIV testing were identified as persons with HIV (PWH) (Table 1).

**Table 1.** Characteristics of Participants With Darkfield Microscopy and PCR results

Characteristic	Total
Total	568
Age (median, IQR)	27 (23, 34)
Sex	
Male	345 (61%)
Female	219 (39%)
HIV status	
Seronegative	404 (71%)
Seropositive	60 (11%)
Missing or unknown	104 (18%)
Darkfield microscopy	
Negative	513 (90%)
Positive	55 (10%)
PCR for STI detection	
* Treponema pallidum*	367 (65%)
* Haemophilus ducreyi*	128 (23%)
Herpes simplex virus	98 (17%)
* Chlamydia trachomatis*	
Non-LGV serovars	35 (6%)
Serovars L1-L3	1 (<1%)
Number of STI etiologies	
None	46 (8%)
One	421 (74%)
Two	95 (17%)
Three	6 (1%)

Missing age: 5.

Missing sex: 4.

Abbreviations: IQR, interquartile range; LGV, lymphogranuloma venereum; PCR, polymerase chain reaction; STI, sexually transmitted infection.

### Participants With Positive Darkfield Microscopy

Overall, 55/568 (10%) of participants had positive DFM results and 54 had additional procedures including collection of clinical data, photography of lesions, and syphilis serologies (Figure 1). Forty-three (80%) of the 54 participants were diagnosed with PS and 11 (20%) were restaged as SS after further review. Of this subset, 44 of 54 (80%) participants had positive RPR tests with a median titer of 1:32, and all had positive TPPA results; 42/54 (78%) had positive TP PCR findings. Among the PS cases, 30 (70%) presented with multiple ulcers. Of those with multiple ulcers, 21 (70%) had positive TP PCR results, 8 (27%) had positive HSV PCR results, and 5 (17%) had both TP and HSV detected.

### Detection of *T. pallidum* by PCR and darkfield microscopy

Of the 568 participants with DFM and qPCR results, 367 (65%) of lesion samples had detectable TP DNA (Figure 1) with a geometric mean copy number of 2381 copies/µL (range 3–1,186,911). DFM was positive in 43/367 (12%) of TP PCR-positive samples and in 12/201 (2%) of TP PCR-negative samples. Compared to TP qPCR, the sensitivity of DFM was 12% and the specificity was 94% using both PS/SS specimens (Table 2). The TP PCR geometric mean values were noted to be higher among DFM-negative swabs than DFM-positive swabs (2707 vs 908 copies/µL).

**Table 2.** Sensitivity and Specificity of Darkfield Microscopy Compared to *T. Pallidum* PCR From All Genital Lesions

	PCR Positive	PCR Negative
Darkfield positive	43 (8)	12 (2)
Darkfield negative	324 (57)	189 (33)

Overall denominator includes participants with primary and secondary syphilis. Sensitivity: (probability of positive darkfield given positive PCR): 12%. Specificity: (probability of negative darkfield given negative PCR): 94%. Includes those reclassified as secondary syphilis (n = 11) after clinical review.

Abbreviation: PCR, polymerase chain reaction.

### PCR Testing for Other STI Pathogens

**Figure 2.**
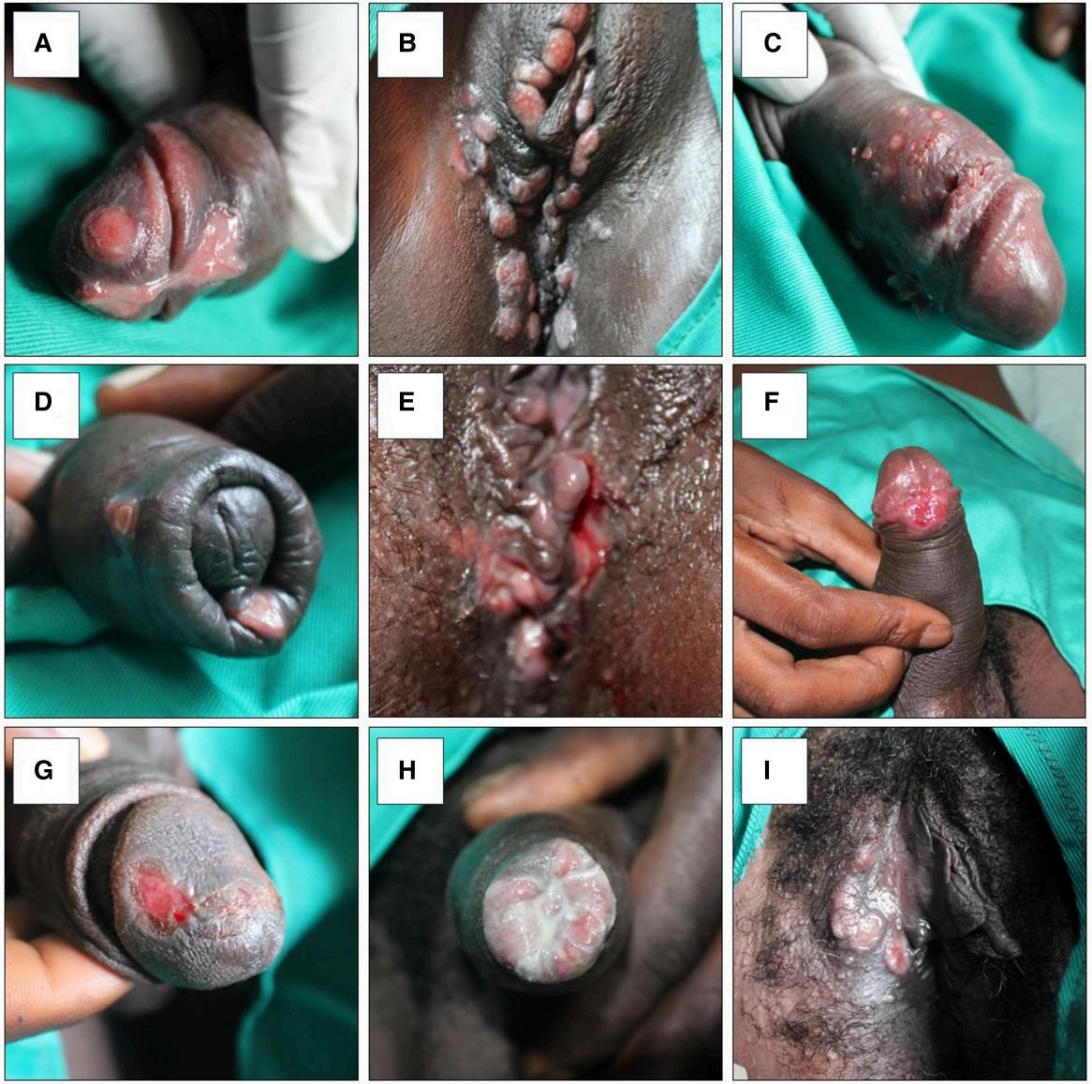
Genital lesions by etiology based on polymerase chain reaction (PCR) results.

From 568 lesion swabs, 128 (23%) samples were identified with HD, 98 (17%) with HSV, and 36 (6%) with CT. Only 1/36 PCR positive for CT had *ompA* sequencing results consistent with LGV serovars L1, L2, or L3, whereas 29 specimens had non-LGV CT serovars and 6 were undetermined. Overall, there were 155 GUD samples (27%) positive for HD, HSV, and/or CT and not TP, and 46 (8%) had negative PCR results for all pathogens tested. Among all 568 participants, 101 (18%) had multiple STIs; among those with positive TP PCRs, 91 of 367 (25%) had coinfections. Twenty-six participants (5%) had both TP and HD, 31 (5%) had TP and HSV, and 28 (5%) had TP and CT (Figure 1). Figure 2 illustrates GUD presentations from participants identified with syphilis (Figure 2*A* and 2*B*), genital herpes (Figure 2*C*), chancroid (Figure 2*D* and 2*E*), LGV (Figure 2*F*), coinfections based on PCR testing (Figure 2*H* and 2*I*) and no etiology (Figure 2*G*).

**Figure 3.**
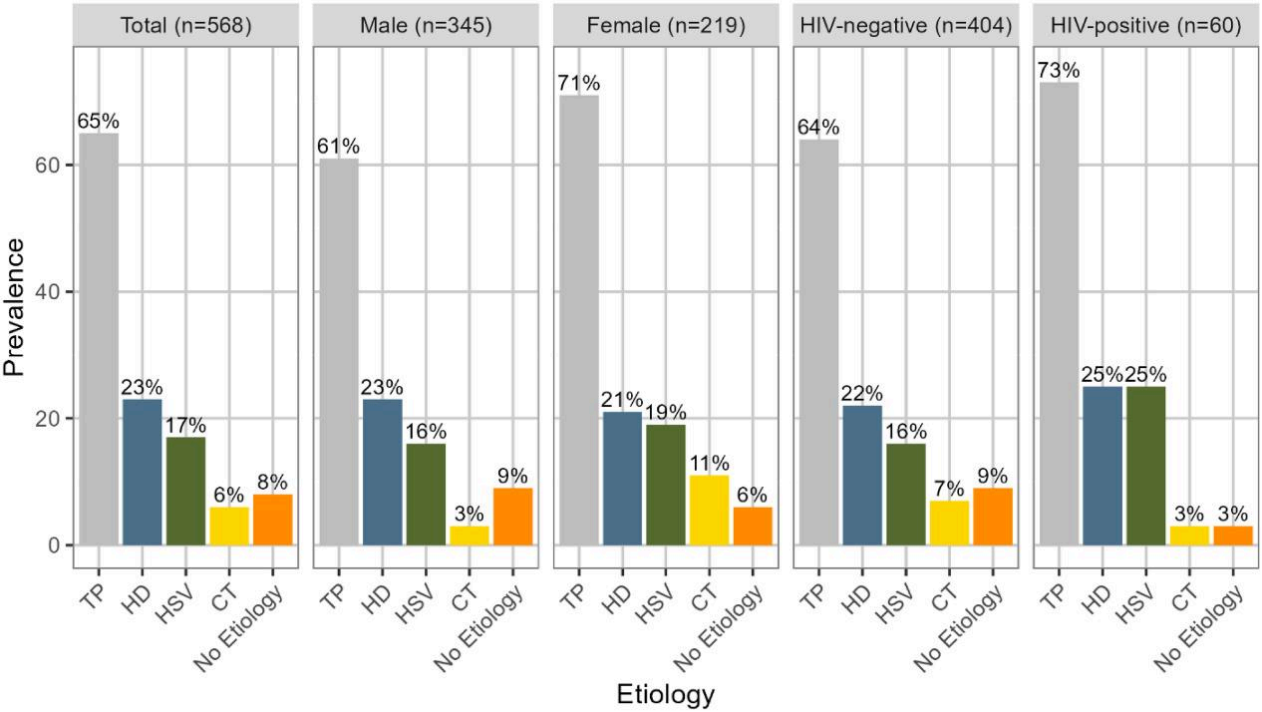
Prevalence of sexually transmitted infections from genital lesions by demographics and human immunodeficiency virus (HIV status).

When comparing male and female participants, HSV and HD had similar distributions (Figure 3), whereas TP and CT were identified in more women than men (TP: 71% vs 61%; CT: 11% vs 3%, respectively). Both TP (73% vs 64%) and HD (25% vs 22%) were slightly more common among PWH compared to HIV-negative participants. We observed a higher proportion of HSV (25%) among PWH compared to HIV-negative participants (16%), but fewer PWH had CT compared to HIV-negative participants (3% vs 7%, respectively).

### External Validation of HD Results Using Allplex PCR

From the 568 samples, 216 (38%) underwent Allplex PCR testing; comparison of singleplex PCR to Allplex results showed substantial concordance (Cohen's kappa 0.78, 89% agreement between assays). The singleplex HD PCR was negative for 7 Allplex HD-positive samples, 5 of which had a high HD DNA concentration (cycle threshold [Ct] range 15–22). The Allplex assay did not detect 16 singleplex HD-PCR positives, all of which had low concentration (Ct range 37–40).

Although samples were not selected for the purpose of validating TP and HSV PCR results, we observed strong concordance in TP PCR results (Cohen's kappa 0.91, 96% agreement) but weak concordance for HSV (Cohen's kappa 0.26, 77% agreement). Differences in HSV results occurred in samples that were positive at higher Ct values (lower concentration) on the Allplex PCR; specifically, the singleplex PCR assay did not detect 51 samples that were positive by the Allplex PCR (50 HSV-2, 1 HSV-1 with Ct ranges 28–39). However, conclusions about relative assay performance for TP and HSV1/2 cannot be drawn because of selection bias.

## DISCUSSION

Chancroid has dramatically declined as a cause of GUD globally [10]. However, we found a high HD positivity rate using 2 different PCR assays and external validation of our results, confirming that 1 in 5 patients presenting with GUD in Malawi had chancroid. This is unexpected given that prior studies demonstrated a decrease in chancroid cases occurring between 1980 and 2014 [17]. TP was the most common pathogen identified, with more than one quarter of syphilis infections identified with other coinfections. We found a low sensitivity for DFM, indicating its suboptimal performance compared to TP qPCR. Thus, our findings underscore the value of molecular methods to assess GUD etiologies among STI patients in LMICs and the need to further investigate chancroid prevalence in Africa.

We identified a high proportion of chancroid in both men and women, PWH, and persons without HIV infection (Figure 1). These results likely confirm a “true” high burden of chancroid in Malawi for 3 reasons. First is that asymptomatic colonization of HD in the genital tract of highly sexually active women such as female sex workers is extremely rare [18]. Second is that, although asymptomatic colonization of HD has been reported in children living in poor sanitary conditions and in areas where yaws is endemic, this does not apply to our study population [16, 17, 19, 20]. Third is that chancroid resulting in GUD versus colonization likely reflects distinct environmental niches for the organism [18]. Interestingly, GUD studies in other African countries have identified minimal chancroid ranging from 0% to 8.6% determined by PCR [18]. A GUD study from Zimbabwe reported that 38.5% were positive for HSV and 16% were positive for TP, whereas no chancroid was detected [19]. Based on the presentation of genital lesions, however, and the identification of HD in 15% with negative PCR results for other STIs (Figure 1), our findings strongly suggest that chancroid is an important cause of GUD in Malawi. Noteworthy is that we enrolled some participants with SS, which could have reduced the proportion of chancroid detected, necessitating a larger surveillance study of genital ulcer cases only.

As part of syndromic GUD management, Malawi has used ciprofloxacin for empiric chancroid treatment [1]. The persistence of HD could be due in part to nonadherence to the multiday treatment or ciprofloxacin resistance. Several HD isolates with intermediate resistance to ciprofloxacin have been reported, but there are few data regarding antimicrobial resistance [21]. In addition, the HD trend could be in part due to infections among female sex workers in the population, which has been reported to drive persistence [18].

Based on our results, the prevalence of TP among persons presenting with GUD in Malawi has increased >10-fold since 2010 [4]. However, because we recruited patients with suspected PS/SS [13], there could have been selection bias resulting in an overestimation of TP prevalence. Nonetheless, these findings emphasize the importance of using POC syphilis tests among patients with suspected syphilis as standard of care in Malawi. We found that PS can present with multiple genital ulcers, even without HSV coinfection. On the other hand, some PS cases were restaged as SS after clinical review, emphasizing the importance of correct staging. In part, the high TP prevalence may be due to the decreasing HSV prevalence, and use of more sensitive TP nucleic acid amplification tests compared to serological testing in prior studies. Overall, our results are consistent with the trend of rising syphilis prevalence worldwide [22–24].

DFM has had reported sensitivities ranging between 75% and 100% for PS and between 58% and 71% for SS [25]. However, we found a much lower sensitivity of DFM (12%) compared to qPCR. Differences may have been due to specimen collection techniques and/or the comparator. We also found that there were higher TP qPCR geometric mean values among samples that were DFM negative, although DFM positivity correlates with high bacterial burdens. Possible explanations may be that more lesion exudate was placed on the slides that were DFM positive, resulting in less sample for qPCR testing from the same swab, or that PCR detected nonmotile organisms. In addition to TP qPCR, a comparison of DFM performance to RPR and TPPA tests would be useful as they are more widely used for syphilis diagnosis.

A systematic review and meta-analysis from sub-Saharan Africa reported that HSV was identified in 57.1% of patients compared to 2.3% for LGV in 2015 [26]. We found a much lower proportion of HSV positives compared to prior studies [4,7,9], which may be explained in part by reported decreases in HSV seroprevalence [27]. However, our limited data available from Allplex PCR testing suggest that our singleplex assay missed low-concentration HSV-1/2 infections. The proportion of HSV ranged from 17% on singleplex to 26% on Allplex; however, these results may be biased since we specifically selected samples for external validation of HD and CT results (not HSV). Nonetheless, our HSV-1/2 prevalence estimates should be viewed as a lower bound and true infection rates are probably higher.

Non-LGV CT serovars were found in a small proportion of specimens, more likely from women with concomitant cervical infections. Only 1 (<1%) specimen had *ompA* genotyping results consistent with serovar L1, L2, or L3, consistent with regional trends [26]. Our findings support the current treatment guidelines, which do not include syndromic treatment for LGV.

We acknowledge several additional limitations. Our results may have been biased because of the recruitment of participants with suspected PS. However, our sample size was quite large and almost all participants with wet/moist ulcers were screened. We had few clinical data on DFM-negative persons because only participants who were DFM positive underwent further data collection [13]. Therefore, we could not conduct other analyses of clinical and laboratory data associated with chancroid and other STIs. Last, our study was conducted in 1 clinic in Lilongwe and may not be generalizable to other African countries.

Our results substantiate the importance of syndromic management for both syphilis and chancroid in Malawi. Similar studies to assess GUD STI etiologies and possible antimicrobial resistance in other parts of Africa are warranted to understand the broader impact of chancroid. In addition, improved control measures for both syphilis and chancroid should be implemented in Malawi—with syphilis screening among all patients with STI and antenatal attendees, provider-assisted partner notification for GUD patients, and the addition of HD to the list of pathogens for investigation in the country's STI surveillance program.

## Supplementary Material

ciaf114_Supplementary_Data

## Supplementary Data

Supplementary materials are available at *Clinical Infectious Diseases* online. Consisting of data provided by the authors to benefit the reader, the posted materials are not copyedited and are the sole responsibility of the authors, so questions or comments should be addressed to the corresponding author.

## Notes

***Acknowledgments.*** The authors thank all the patients who participated in this study and the Bwaila STI clinic and Dermatology clinic teams. They are also thankful to the UNC Project Malawi STI research team for their support. They thank the following people who supported this research project in various ways, listed by institution: Ellena Mlolo (UNC Project Malawi), Grace Kaphela (UNC Project Malawi), Zondwayo Tembo (UNC Project Malawi), Boneface Musisi (UNC Project Malawi), Sandress Lupunga (UNC Project Malawi), Lloyd Singogo (UNC Project Malawi), Mary Kacheyo (UNC Project Malawi), Edith Ngoma (UNC Project Malawi), Cecilia Massa (UNC Project Malawi), Chazirwa Nyasulu (UNC Project Malawi), Annie Kachigamba (Bwaila District Hospital), Margaret Shaba (Bwaila District Hospital), Henry Nkhata (Lighthouse Trust), Cliff Jabulo (Lighthouse Trust), Chikondano Makaka (Lighthouse Trust), Alex Tembo (Lighthouse Trust), Theresa Namasani (Lighthouse Trust), Vincent Chingale (Lighthouse Trust), Richard Maganga (Lighthouse Trust), Rapson Chidothe (Bwaila Dermatology clinic), Dingase Ngongonda (Bwaila Dermatology clinic), and Derlings Duma (Bwaila Dermatology clinic).

***Financial support.*** Research reported in this publication was supported by the National Institute of Allergy and Infectious Diseases of the National Institutes of Health under award numbers U19 grant (U19AI144177 to J. D. R.) and U19 RFA-AI-18-005. This work also was supported, in part, by the Bill and Melinda Gates Foundation (INV-036560 to A. C. S.).
