# Supplementary material for: High Prevalence of Haemophilus ducreyi Among Patients With Suspected Primary syphilis in Malawi, 2019–2022
Source: Clin Infect Dis. 2025 Mar 17;82(3):e604–11. doi: 10.1093/cid/ciaf114 (PMC13016763; doi:10.1093/cid/ciaf114)
Supplement: ciaf114_Supplementary_Data [file ciaf114_supplementary_data.docx]

**Supplement 1**

**PCR assay for** ***T. pallidum* quantification**

Samples underwent quantitative PCR following a previously described method^1^. Serially diluted linearized plasmid containing the *polA* gene was amplified alongside samples to construct a standard curve, which was then used to determine the number of *T. pallidum* genome copies per uL of each DNA sample.

**PCR assays for *H. ducreyi*, HSV1/2, *C. trachomatis* detection**

All PCR assays were performed on a Bio-Rad CFX384 qPCR system (Bio-Rad, Hercules, CA, US), using FastStart Universal Probe Master (ROX) (Roche, Indianapolis, IN, US) and cycling conditions consisting of 1 min at 50C, 10min at 95C, and 45 cycles of 15sec at 95C, and 1 min at 60C. Positives were called for isolates with Ct values <40.

Targets, sequences, reaction concentrations, and references for all primers and probes used are included in **Table S1**. In some cases, fluorophores were altered compared to the original published assays.

**Table S1.** Primers, probes, and reaction conditions for singleplex HSV1/2, *H. ducreyi,* and LGV PCR assays used in this study.

| **PCR target** | **Primer/Probe** | **Reaction concentration** | **Sequence 5'-3'** | **Reference** |
| --- | --- | --- | --- | --- |
| glycoprotein D gene (*gD*) | Ct Fwd | 900nM | GGATAACTCTGTGGGGTATTCGCCT | Chen et al. 2012.^2^ |
|  | Ct Rev | 900nM | ACGCCTGAGATCTCCAAACTAGT |  |
|  | Ct Probe FAM | 200nM | FAM-TAGACCCTTTCCGAGCATCACTAACTGTTG-BHQ1 |  |
| *pmpH* | HSV Fwd | 800nM | CCCCGCTGGAACTACTATGACA | Chen et al. 2007.^3^ |
|  | HSV Rev | 800nM | GCATCAGGAACCCCAGGTTA |  |
|  | HSV Probe CY5 | 200nM | CY5-TTTAGCGCCGTCAGCGAGG-BHQ2 |  |
| *16S rRNA* | HD Fwd | 600nM | ACATCCATAGAAGAACTCAGAGATGA | Glatz et al. 2014.^4^ |
|  | HD Rev | 600nM | TTGAGTTCCCATCAYTACATGCT |  |
|  | HD Probe HEX | 200nM | HEX-GTGCCTTCGGGAACTATGTGACAGGT-BHQ1 |  |

All assays included no-template (negative) controls, as well as commercially available positive controls as described in **Table S2.**

**Table S2.** Positive controls used in this study.

| **Pathogen** | **Positive Control Used** | **Company** | **Catalog Number** |
| --- | --- | --- | --- |
| Herpes simplex virus I | AMPLIRUN® HERPES SIMPLEX 1 DNA CONTROL | Vircell | MBC023 |
| Herpes simplex virus II | AMPLIRUN® HERPES SIMPLEX 2 DNA CONTROL | Vircell | MBC024 |
| *Chlamydia trachomatis* | AMPLIRUN® CHLAMYDIA TRACHOMATIS DNA CONTROL | Vircell | MBC012 |
| *Haemophilus ducreyi* | AMPLIRUN® HAEMOPHILUS DUCREYI DNA CONTROL | Vircell | MBC021-R |

**Serovar determination using *ompA* sequencing**

After amplification and sequencing as described in the manuscript, bi-directional Sanger sequences were trimmed, assembled, and genotyped using Geneious v2023.0.1. Consensus sequences, as well as reference *ompA* sequences available from NCBI and the recombinant L2b/D-Da *ompA* sequence ^5^, were aligned. Serovars were called based on similarity to reference. All consensus sequences were exact matches to their respective serovar reference sequences, with the exception of two strains that had two variants each (samples TPVMW190T, TPVMW6257, both with 98.7% identity).

**GenBank Accession Number for Nucleotide sequences**

BankIt2944548 TPVMW313C         PV453707

BankIt2944548 TPVMW527V         PV453708

BankIt2944548 TPVMW592Y         PV453709

BankIt2944548 TPVMW755L-73      PV453710

BankIt2944548 TPVMW2925         PV453711

BankIt2944548 TPVMW679B         PV453712

BankIt2944548 TPVMW685C-26      PV453713

BankIt2944548 TPVMW737F-31      PV453714

BankIt2944548 TPVMW770H-43      PV453715

BankIt2944548 TPVMW6902         PV453716

BankIt2944548 TPVMW654T-21      PV453717

BankIt2944548 TPVMW654T-23      PV453718

BankIt2944548 TPVMW3358         PV453719

BankIt2944548 TPVMW735B-37      PV453720

BankIt2944548 TPVMW685C-24      PV453721

BankIt2944548 TPVMW356P         PV453722

BankIt2944548 TPVMW407V         PV453723

BankIt2944548 TPVMW654T-22      PV453724

BankIt2944548 TPVMW565F         PV453725

BankIt2944548 TPVMW655V         PV453726

BankIt2944548 TPVMW685C-25      PV453727

BankIt2944548 TPVMW2821         PV453728

BankIt2944548 TPVMW134U         PV453729

BankIt2944548 TPVMW118A         PV453730

BankIt2944548 TPVMW124Y         PV453731

BankIt2944548 TPVMW142A         PV453732

BankIt2944548 TPVMW222A         PV453733

BankIt2944548 TPVMW535V         PV453734

BankIt2944548 TPVMW048P         PV453735

BankIt2944548 TPVMW255S         PV453736

BankIt2944548 TPVMW079S         PV453737

BankIt2944548 TPVMW150A         PV453738

BankIt2944548 TPVMW735B-36      PV453739

BankIt2944548 TPVMW4657         PV453740

BankIt2944548 TPVMW278A         PV453741

BankIt2944548 TPVMW422T         PV453742

BankIt2944548 TPVMW190T         PV453743

BankIt2944548 TPVMW6257         PV453744

**References**

1. Luthra A, Montezuma-Rusca JM, La Vake CJ, et al. Evidence that immunization with TP0751, a bipartite Treponema pallidum lipoprotein with an intrinsically disordered region and lipocalin fold, fails to protect in the rabbit model of experimental syphilis. PLoS Pathog. 2020;16(9):e1008871. Published 2020 Sep 16. doi:10.1371/journal.ppat.1008871
2. Chen, CY., Ballard, R.C. (2012). The Molecular Diagnosis of Sexually Transmitted Genital Ulcer Disease. In: MacKenzie, C., Henrich, B. (eds) Diagnosis of Sexually Transmitted Diseases. Methods in Molecular Biology, vol 903. Humana Press, Totowa, NJ. <https://doi.org/10.1007/978-1-61779-937-2_6>
3. Chen, Cheng-Yen*; Chi, Kai-Hua*; Alexander, Sarah†; Martin, Iona M. C.†; Liu, Hsi*; Ison, Cathy A.†; Ballard, Ronald C.*. The Molecular Diagnosis of Lymphogranuloma Venereum: Evaluation of a Real-Time Multiplex Polymerase Chain Reaction Test Using Rectal and Urethral Specimens. Sexually Transmitted Diseases 34(7):p 451-455, July 2007. | DOI: 10.1097/01.olq.0000245957.02939.ea
4. M. Glatz, N. Juricevic, M. Altwegg, S. Bruisten, P. Komericki, S. Lautenschlager, R. Weber, P.P. Bosshard, A multicenter prospective trial to asses a new real-time polymerase chain reaction for detection of Treponema pallidum, herpes simplex-1/2 and Haemophilus ducreyi in genital, anal and oropharyngeal ulcers, Clinical Microbiology and Infection, Volume 20, Issue 12, 2014, Pages O1020-O1027, ISSN 1198-743X, <https://doi.org/10.1111/1469-0691.12710>
5. Vítor Borges, Joana Isidro, Cristina Correia, Dora Cordeiro, Luís Vieira, Zohra Lodhia, Cândida Fernandes, Ana Maria Rodrigues, Jacinta Azevedo, João Alves, João Roxo, Miguel Rocha, Rita Côrte-Real, Cristina Toscano, Maria Ana Pessanha, Israel Nissan, Shlomo Pilo, Efrat Rorman, Zeev Dveyrin, Yossi Paitan, Haim Paran, Gal Wagner-Kolasko, Jennifer Beirnes, Suzanne Gibbons, Alberto Severini, Maria José Borrego, João Paulo Gomes, Transcontinental Dissemination of the L2b/D-Da Recombinant *Chlamydia trachomatis* Lymphogranuloma venereum (LGV) Strain: Need of Broad Multi-Country Molecular Surveillance, *Clinical Infectious Diseases*, Volume 73, Issue 4, 15 August 2021, Pages e1004–e1007, <https://doi.org/10.1093/cid/ciab067>
